# EOAD‐Signature Atrophy Predicts Progression to Dementia in Patients with Early‐onset MCI due to Alzheimer’s Disease

**DOI:** 10.1002/alz70862_109979

**Published:** 2025-12-23

**Authors:** Thiago Paranhos, Yuta Katsumi, Michael Brickhouse, Ryan Eckbo, Alexander Zaitsev, Anna Du, Ani Eloyan, Renaud La Joie, Kelly N. Nudelman, Tatiana M. Foroud, Jeffrey L. Dage, Maria C. Carrillo, Gil D. Rabinovici, Liana G. Apostolova, Brad C. Dickerson, Alexandra Touroutoglou

**Affiliations:** ^1^ Frontotemporal Disorders Unit, Department of Neurology, Massachusetts General Hospital and Harvard Medical School, Boston, MA USA; ^2^ Frontotemporal Disorders Unit and Massachusetts Alzheimer’s Disease Research Center, Department of Neurology, Massachusetts General Hospital and Harvard Medical School, Boston, MA USA; ^3^ Department of Biostatistics, Brown University, Providence, RI USA; ^4^ Department of Neurology, University of California, San Francisco, San Francisco, CA USA; ^5^ Department of Neurology, Indiana University School of Medicine, Indianapolis, IN USA; ^6^ Department of Medical and Molecular Genetics, Indiana University School of Medicine, Indianapolis, IN USA; ^7^ Medical & Scientific Relations Division, Alzheimer's Association, Chicago, IL USA; ^8^ Department of Radiology and Imaging Sciences, Center for Neuroimaging, Indiana University School of Medicine, Indianapolis, IN USA

## Abstract

**Background:**

Prognostic risk stratification for patients at the mild cognitive impairment (MCI) stage of early‐onset Alzheimer’s disease (EOAD) would allow professionals and loved ones to make better‐informed medical and life planning decisions. While research including our own (Bakkour, Morris, Dickerson, 2009) has demonstrated the prognostic value of MRI‐based measures of brain structure in late‐onset amnestic AD, its utility for predicting progression to dementia in EOAD remains unclear. Here, we measured the magnitude of cortical atrophy within our recently described EOAD signature regions (Touroutoglou et al. 2023) in patients with EOAD at the MCI stage (*N* = 130) recruited in LEADS. The main goal of the study was to evaluate the utility of this measure as a predictor of time to subsequent progression to dementia. Our second goal was to examine the independent or synergistic contributions of EOAD signature of atrophy and standard clinical severity measures used in clinical trials.

**Method:**

For each patient, we measured the time between baseline visit and subsequent visit at which progression to mild dementia was documented or last observation. Baseline cortical atrophy was measured as *W*‐scores (i.e., *Z*‐scores adjusted for age and sex relative to a sample of healthy controls) in the EOAD signature. Baseline clinical severity was quantified with the Clinical Dementia Rating Sum‐of‐Boxes scores (CDR‐SB). Simple and multivariable Cox regression models examined the relationship between atrophy in EOAD signature, baseline CDR‐SB, and the likelihood of progression to dementia.

**Result:**

Greater baseline atrophy in the EOAD signature predicted higher risk of progression to dementia (hazard ratio = 1.2, 95% CI 1.1‐1.3) and provided additive value to the CDR‐SB (hazard ratio: 2.1, 95% CI:1.7‐2.8) in predicting progression.

**Conclusion:**

These findings point to the role of EOAD MRI signature as an imaging biomarker to guide prognostication for patients with EOAD and their families and to inform the design of clinical trials.

